# Novel Corona Virus (COVID-19): Assessing Prevalence of SARS-CoV-2 and Infection

**DOI:** 10.30476/DENTJODS.2021.89631.1425

**Published:** 2022-09

**Authors:** Ehsan Hooshyar, Sarah Hosseini

**Affiliations:** 1 Dept. of Periodontology, Faculty of Dentistry, Urmia University of Medical Sciences, Urmia, Iran; 2 Private Practice, Urmia, Iran

**Keywords:** COVID-19, Dentist, SARS-COV-2, Iran

## Abstract

**Statement of the Problem:**

The World Health Organization (WHO) declared severe acute respiratory syndrome coronavirus 2 a pandemic on March 11, 2020. Corona virus disease 2019 (COVID-19) is commonly transmitted from human-to-human via close contacts and touching surfaces. Reports indicated that many medical staff got infected on working with infected individuals. Likewise, dentists are at a higher risk for the virus transmission due to close proximity to patients and the nature of dental procedures. Despite all of the protections and disinfections, there were some reports of infected dentists.

**Purpose:**

In this study, we evaluated the prevalence of infected dentists and the rate of using protection protocols by them in Iran.

**Materials and Method:**

This survey was a cross-sectional descriptive and web-based study in which a questionnaire designed and uploaded on Google forms. The link of the form was shared among dentists in Iran via some social media groups and personal messages.

**Results:**

A total of 945 dentists participated in this survey. A higher proportion of participants had their own private practice. About one third reported fatigue, malaise, or headaches since the COVID-19 got epidemic in Iran. Most of the respondents had provided emergency and elective dental treatments, used face shields, and surgical masks. The 26.3% of respondents were positive for COVID-19.

**Conclusion:**

According to our analysis, about 26% of dentists had been infected with COVID -19 and most of them reported that they used the CDC’s currently recommended infection prevention and control procedures in dental offices. However, the prevalence of infection was higher than prevalence of infection in the whole population of Iran (approximately 1%) and it showed that dentists were at high risk despite using infection control and personal protection equipment (PPE).

## Introduction

On March 11, 2020, World Health Organization (WHO) declared a pandemic of severe acute respiratory syndrome coronavirus 2 (SARS-CoV-2), the virus that causes coronavirus disease 2019 (COVID-19) [ 1 ]. SARS- CoV-2 belongs to the beta coronavirus genus, which includes the SARS-CoV-1 coronavirus, Middle East Respiratory Syndrome (MERS) coronavirus, and two other human corona viruses, HCoV-OC43 and HCoV-HKU1. SARS-CoV-1 and MERS coronavirus cause severe infections with about 9% and 36% mortality rates respectively but their transmission remained limited [ 2 ]. Recent study showed that COVID-19 is more infectious than SARS-CoV and MERS-CoV [ 3 ]. COVID-19 is commonly transmitted from human-to-human via handshake, oral and nasal secretions, or droplets, and touching the surfaces [ 4 - 5 ]. The average incubation period of COVID-19 is about 4 days to two weeks [ 6 ]. The infected person usually represents upper respiratory tract infection and complaints of high-grade fever, dry cough, and dyspnea [ 7 ].

 Evidence shows that many medical staff s have got infected on working with infected individuals [ 8 ]. Dental clinics not only are not exception from this possibility of transmission and acquisition of the disease but also have a higher risk for the virus transmission due to close proximity to patients and the nature of dental procedures [ 9 ]. The cross-infection in dentistry is considerably high, since aerosols and splatters contribute to increased risk, especially during aerosol-generating dental procedures (AGDPs) [ 10 - 12 ]. Respiratory viruses can spread through three ways: contact (direct or indirect), droplets, and aerosols [ 13 - 14 ]. Contact transmission means direct transmission of virus from infected person to susceptible person, for example from infected hands or indirect virus transmission via intermediate objects (fomite). Airborne transmission can occur via aerosols and droplets. The average size between the large droplets and small aerosols is 5µm, although this differs among studies, ranging up to 12µm [ 15 - 18 ]. 

Micik *et al.* [ 19 ]’s study introduced the words “aerosol” and “splatter” in dental environment. They declared that aerosols are particles less than 50µm in diameter. These particles are able to stay airborne due to their small size for a long time before they settle on surfaces or enter respiratory tract. Smaller particles of aerosols (with 0.5-10µm in diameter) can penetrate and reside in the smaller airway tracts. Splatter was defined as a particle with the size of greater than 50µm in diameter. These particles are too large to become airborne [ 20 - 23 ]. The greatest airborne infection threat in dentistry is from aerosols (particles less than 50μm in diameter) because they can stay airborne and enter respiratory system [ 24 - 25 ]. 

A study done in China has reported that the cell receptor for COVID-19 infection, angiotensin-converting enzyme II (ACE 2), is highly expressed in the oral mucosa particularly in the epithelial cells of the tongue [ 26 ]. These findings point out that the oral cavity is one of the high-risk transmitters of SARS-CoV-2 infection.

Due to incubation period of COVID-19 and the high potential of infection spread, use of personal protection equipment (PPE), like mask, gloves, and eye protection (goggles), decreases the risks of infection [ 27 ]. It is recommended to use particulate respirators like N-95 mask for treatment of suspected patients of COVID-19. Otherwise, a surgical mask must be used during all dental procedures if there is less than 1-meter distance between dental healthcare workers and the patients [ 28 ]. In addition to PPE, American Dental Association (ADA) recommends dentists to take some key steps including: taking patient’s recent travel history, evaluating symptoms and signs of respiratory infections, measuring the body temperature of patient, using mouthwash 1% hydrogen-peroxide before any dental treatment, apply rubber dam and high volume evacuation, cleaning and sanitizing every surfaces with public contacts, like door handles, chairs, and wash rooms [ 29 ].

Despite all of the protections and disinfections, there are some reports of infected dentists. In this study, we evaluated the prevalence of infected dentists and the rate of using protection protocols by them in Iran using web-based questionnaire.

## Materials and Method

This survey was a cross-sectional descriptive and web-based study in which a questionnaire designed and uploaded on Google forms, from 27 November 2020 to 9 December 2020. Then the link of the form was shared among dentists in Iran via some social media groups and personal messages. The survey included 25 questions. The questions were in Persian (Iran’s official language). Demographic questions included age, gender, primary practice location/locations, underlying disease, medical ID number, and specialty. Then there were some COVID-19 related questions (signs, symptoms, tests, contacts, social distancing, etc.). In addition, some questions were designed to evaluate the rate of following the protocols for PPE and environmental infection control. All data collected through the answers analyzed by SPSS (V.22) and reported as results.

## Results

At the time of survey, there were about 39,000 dentists in Iran that include specialists and general dentists [ 30 ]. A total of 20,430 of the dentists were male and 18,451 were female. Most of dentists were in the age of 25-30 years old. The number of general dentists and specialists were 34,005 and 4,714 respectively. All the dentists who had medical ID number were participated in the survey.

### Participant characteristics

A total of 945 dentists participated in this web-based survey, from 27 November 2020 through 9 December 2020. The mean age of participants was 40 years old (standard deviation=11.69). The 56.8% (n=537) of respondents were female and 43.2% (n=408) were male and most of them had focused on general dentistry (82.2%). A higher proportion of participants (86.3%) had their own private practice ( Table 1).

**Table 1:** The prevalence of symptoms and activities done by dentists

Symptoms/Activity	Percent
Symptoms	
Fatigue and malaise	27.3
Headache	24.1
Sore throat	22.9
Muscle soreness	18.4
Fever	16.5
New loss of taste or smell	14.6
Chill	14.3
Diarrhea	12.1
Dry cough	10.5
Shortness of breath or difficulty breathing	6.7
Nausea and vomiting	6
others	1.5
No symptoms	47.9
**Activity**	
Provided emergency oral health care	79.7
Provided elective oral health care	72.1
Visiting a patient	8.6
Met in person with anyone outside your household	47
Met with a group of 10 or more people in a social setting	26
Attended any public event with 50 or more people	10.2
Traveled via taxi, ride share, or public transportation	23.2
Had contact with anyone with suspected or confirmed COVID-19	27

### Signs and Symptoms among Participants

The participants were asked whether they had any symptoms like fever, diarrhea and sore throat, regardless of infected with COVID-19 or not, from 20 February 2020 until 9 December. Most had no symptoms but 27.3% re-ported fatigue or malaise and 24.1% reported headaches ( Table 2).

**Table 2:** The prevalence of medical conditions and specialty and dental practice type of participants

Parameter	Number (%)
Medical condition	
Obesity	105 (28.5)
Smoking	51 (13.8)
Cardiovascular disease	51 (13.8)
Autoimmune disease	42 (11.4)
Diabetes	24 (6.5)
Asthma	18 (4.9)
Liver disease	6 (1.6)
Immunosuppress disease	3 (0.8)
COPD*	3 (0.8)
Other	66 (17.9)
**Specialty**	
General Dentist	783 (82.86)
Pedodontist	30 (3.17)
Oral medicine	27 (2.86)
Periodontist	18 (1.90)
Endodontist	15 (1.59)
Oromaxillofacial radiologist	15 (1.59)
Orthodontist	12 (1.27)
Oromaxillofacial surgeon	12 (1.27)
Esthetic dentistry	12 (1.27)
Oromaxillofacial pathology	12 (1.27)
Prosthodontist	9 (0.95)
**Dental practice type**	
Private practice	816 (86.3)
Hospitals	57 (6)
City or country healthcare centers	177 (18.7)
University or academic-therapeutic centers	114 (12.1)

* Chronic Obstructive Pulmonary Disease

### Activity

Among all respondents, most of them had provided emergency and elective dental treatments (79.7% and 72.1 % respectively). However, there were few reports of gathering in groups with more than 50 people ( Table 2).

### Dental Practice and Infection Control and PPE

It is reported that corona virus can be transmitted through aerosols and droplets [ 2 ], the participants were questioned, whether they provided aerosol generating dental procedures (AGDP) since 20 February 2020 until 9 December, and 87.8% of answers were yes.

The participants were asked about the protocols they considered for decreasing the rate of infection and transmission. Most of the dentists used face shields and surgical masks (89.5% and 79.7% respectively). Some used mouth rinse before treatment, rubber dam, or sterile gown (47.9%, 5.7%, and 74.3% respectively). A few reported powered air-purifying respirator (PAPR) and N100 masks ( Table 3).

**Table 3:** The rate of infection control ways and PPE among dentists

Infection control and PPE	Percent
Goggles	64.4%
Face shield	89.5%
FFP2 (N95) or FFP3 (N99)	74.6%
3 layer surgical mask	79.7%
Sterile gown	74.3%
Plastic shoes covering	37.8%
Air reinforced filters	34%
Rubber dam	5.7%
Mouth rinse before procedure	47.9%
Others (PAPR, opening windows, etc.)	6.6%

PPE: Personal Protection Equipment, FFP: Filtering Face Piece, PAPR: Power Air Purifying Respirator

Approximately half of dentists (51.5%) changed their masks at the end of day and there were just 36 dentists who changed their masks patient by patient.

Dentists were asked whether their assistants used same personal protection equipment and had social distancing with them. A total of 79.1% claimed that their assistants had the same PPE and 69.9% had social distancing with their assistants.

It was questioned that how many patients were visited and treated by participants every month by average. The mean number of patients was assessed 84 for each month (standard deviation= 115.116) that means approximately three patients every day.

### Approved or possible COVID-19 among dentists

Among participants, 34.6% (n=327) reported that they had been tested for COVID-19 with at least one testing types. There were three types of COVID-19 testing methods in Iran when this survey was done: PCR, Serology, and CT-scan. A total of 228 (69.7%) of dentists were tested by PCR, and 81 (24.7%) and 114 (34.8%) by CT-scan and serology, respectively. The 76.14% of respondents who had been tested for COVID-19 were positive for COVID -19, which was 26.3% of all participants (945). About 51.8 % (n=129) of positive COVID-19’s were female and 48.2% (n=120) were male. Most approved corona virus disease patients were at age <30 years old ( Table 4).

**Table 4:** The prevalence of COVID-19 among age groups

Age group of positive COVID (years old)	Percentage (number)
< 30	33.7 (n=84)
30-39	26.5 (n=66)
40-49	21.6 (n=54)
≥ 50	18.2 (n=45)

Most of the tests were done in November, when there was the third pick wave of coronavirus in Iran.

A total of 36.14% of participants who had been positive for COVID-19 reported that their contamination route was the result of dental practices. About 17% of participants reported that they had received a diagnosis of possible SARS-CoV-2 infection by a physician.

There were also a question that asked the dentists whether they had attended courses about COVID-19 or not, and 27% answers were positive. As dentists who died of SARS-COV-2 were not considered in our survey, a question was designed to get the approximate number of expired dentists. We had lost 33 dentists and if this was added to infected dentists, 29.8% of dentists were infected by SARS-COV-19 and 3.5% were died as a result of coronavirus infection.

## Discussion

The study assessed the rate of COVID-19 infection among Iranian dentists. 26.3% of participants have or have had COVID-19 and there were 33 dentists who died in result of the infection. These results differed from studies done in US [ 30 ], Netherlands [ 31 ], China [ 32 ], Seattle, and Washington [ 33 ] (0.9%, 0.9%, 1.1%, and 5.3% respectively).

This rate of infection among dentists was higher than the infection prevalence of the whole Iran’s population [ 34 ]; this difference may be the result of high exposure of dentists to aerosols. However, the mortality rate is lower (4.66% among Iranian population). This lower rate could be the result of higher knowledge about corona virus, faster treatment approach, or continuous exposure to virus that could cause immune response in their bodies. A total of 99.7% of dentists have reported that they were using enhanced infection protection and control procedures. This high rate of infection control might manifest the current CDC guidance about enhancing PPE [ 35 ].

The survey sample might also be subjected to selection bias, leading to underestimate of COVID-19 prevalence because dentists who have expired or been confined in hospitals with COVID-19, for example, could not or might have been less interested to participate, although we tried to decrease this bias by asking participants about the dentists who died due to COVID-19.

Another bias can be social desirability bias because the participants might have reported higher levels of social distancing and infection control [ 36 ].

A total of 62.4% of infected participants did not know the probable source of their infection while 40% reported they had infected through dental procedures. Although it is not possible to recognize the exact way of infection, while most of the dentists had attended nonclinical activities that can be the source of COVID-19 too.

## Conclusion

This survey was conducted to evaluate the prevalence of COVID-19 infection among dentists and infection control protocols in Iran. Based on our analysis, about 26% of dentists had been infected with COVID-19 and most of them reported that they used the CDC’s current guidance for infection control in dental procedures. However, the prevalence of infection was higher than prevalence of infection in the whole population of Iran (approximately 1%) and it showed that dentists were at high risk despite using infection control and personal protection equipment (PPE).

## Acknowledgment

The authors gratefully acknowledge Urmia University of Medical Sciences for supports.

## Conflicts of Interest

The authors declare that they have no conflict of interest.
